# Steroid Profiles and Precursor-to-Product Ratios Are Altered in Pregnant Women with Preeclampsia

**DOI:** 10.3390/ijms252312704

**Published:** 2024-11-26

**Authors:** Olivia Trummer, Christina Stern, Sharmaine Reintar, Karoline Mayer-Pickel, Mila Cervar-Zivkovic, Ulrich Dischinger, Max Kurlbaum, Berthold Huppertz, Herbert Fluhr, Barbara Obermayer-Pietsch

**Affiliations:** 1 Department of Internal Medicine, Division of Endocrinology and Diabetology, Medical University of Graz, 8036 Graz, Austria; sharmaine.reintar@medunigraz.at (S.R.); barbara.obermayer@medunigraz.at (B.O.-P.); 2 Department of Obstetrics and Gynecology, Division of Obstetrics, Medical University of Graz, 8036 Graz, Austria; christina.stern@medunigraz.at (C.S.); karoline.pickel@medunigraz.at (K.M.-P.); mila.cervarzivkovic@medunigraz.at (M.C.-Z.); herbert.fluhr@medunigraz.at (H.F.); 3 Department of Internal Medicine I, Division of Endocrinology and Diabetes, University Hospital Würzburg, 97080 Würzburg, Germany; dischinger_u@ukw.de (U.D.); kurlbaum_m1@ukw.de (M.K.); 4 Core Unit Clinical Mass Spectrometry, University Hospital Würzburg, 97080 Würzburg, Germany; 5 Division of Cell Biology, Histology and Embryology, Gottfried Schatz Research Center, Medical University of Graz, 8010 Graz, Austria; berthold.huppertz@meduni-graz.at

**Keywords:** preeclampsia, steroids, steroid metabolism, steroid-metabolizing enzymes, precursor-to-product ratios

## Abstract

Steroid hormone imbalance is associated with the pathogenesis of preeclampsia. However, affected enzymes of steroid metabolism and gene and protein expression in serum and placenta have not been elucidated yet. We aimed to investigate steroid hormone profiles and precursor-to-product ratios in preeclamptic women compared to women with healthy pregnancy (controls) to identify potentially affected steroid hormones and their metabolizing enzymes. Also, we aimed to investigate whether the mRNA expression of these enzymes is different between the study groups and whether levels of serum mRNA expression reflect postnatal placental protein expression. Serum levels of 14 steroid hormones were measured at eight time points throughout pregnancy in nine preeclamptic women and 36 controls. Serum mRNA expression of selected steroid-metabolizing enzymes was assessed, and their protein expression was analyzed in additional nine preeclamptic women. Mean levels of sex steroid and corticosteroid hormones were significantly altered in preeclamptic women. Precursor-to-product ratios of 5α-reductase, aromatase and 11β-hydroxysteroid dehydrogenase 1 were significantly increased, those of steroid 17α-hydroxylase, 17β-hydroxysteroid-dehydrogenase, steroid 11β-hydroxylase and 11β-hydroxysteroid dehydrogenase 2 were significantly decreased. Serum mRNA expression and placenta protein expression were comparable between the groups. Results contribute to understanding the heterogeneity of preeclampsia and can thus promote future research in personalized medicine.

## 1. Introduction

Preeclampsia is a complex pregnancy-specific multiorgan disorder, affecting 2% to 8% of all pregnancies [1,2]. Despite advances in feto-maternal management, preeclampsia is still a major cause of maternal and neonatal morbidity and mortality worldwide, especially in developing countries [1]. Manifestation of preeclampsia is associated with serious long-term neonatal and maternal complications such as fetal growth restriction and potential iatrogenic preterm delivery and increased maternal risk of cardiovascular morbidity and mortality later in life [3]. Hallmark features of preeclampsia are hypertension and endothelial dysfunction, leading to widespread damage to the liver and the kidneys. Termination of pregnancy is still the only curative treatment for preeclampsia [1,4,5,6]. Although the clinical symptoms of patients with preeclampsia do not appear until after 20 weeks of gestational age (WGA), it is widely believed that the molecular events leading to its onset have already occurred early in pregnancy [7]. The exact mechanisms of preeclampsia are still unknown, but several systemic changes such as angiogenic imbalance, oxidative stress and exaggerated systemic inflammation have been proposed [1,8,9]. 

Women with preeclampsia show dysregulated hormone production, which might contribute to the pathogenesis of the disease and reflect underlying mechanisms of placental and vascular function [10,11].

Potential biomarkers for preeclampsia largely originate from organs that are involved in its pathogenesis. As such, the placenta and endothelial cells represent potential sources of biomarkers that may be present as circulating acids, metabolites and proteins in maternal blood [4]. 

Normal pregnancy is characterized by a significant increase of progesterone, estrogens, cortisol and aldosterone, while dehydroepiandrosterone sulfate (DHEAS) is slightly decreased. These physiological adaptions are required for the development of the fetus and maternal physiology [12,13,14]. Imbalances of steroid hormones, especially androgens, have been associated with the pathogenesis of preeclampsia [10,11]. These imbalances relate, in particular, to the steroid hormone classes progestogens, gonadal steroids and glucocorticoids and their precursor cholesterol that are required for the successful establishment and maintenance of pregnancies, the proper development of the fetus and the onset and progression of parturition [15,16,17]. 

The synthesis of steroid hormone begins with the conversion of cholesterol as a substrate during which structurally related products are formed via a series of enzymatic reactions. This process is tightly regulated by the tissue- and cell-specific expression of steroidogenic enzymes [18]. The steroid precursor-to-product ratios in maternal serum can be used as surrogate for the enzymatic activity in steroid hormone metabolism originating from maternal glands, the decidua, the placenta and/or the fetus [19]. Therefore, changes in precursor-to-product ratios may indicate changes in the enzymatic activity caused by, e.g., inhibition of activity or downregulation of enzymes on a molecular level. In pregnant women without complications, steroidogenesis physiologically increases approximately two- to threefold during pregnancy [16].

During pregnancy, the placenta and fetus participate in the biosynthesis and metabolism of steroids. As a developing organ, the placenta is constantly adapting to the maternal environment; this adaptation includes not only the structure but also the transcriptome [20]. Thereby, RNA molecules are actively secreted or released into the maternal circulation by cells undergoing necrosis or apoptosis and may have important roles in feto-maternal signaling [21,22]. Methods to pinpoint regulatory changes in affected enzymes involve the investigation of circulating nucleic acids, including RNAs. Although circulating nucleic acids are not yet recommended in clinical diagnostic practice [23], they have the potential to be used as biomarkers and thus represent a promising minimally invasive option for diagnosis in the future [23].

Aim of the present study was to investigate serum steroid hormone profiles and their precursor-to-product ratios in preeclamptic women compared to women with healthy pregnancy (controls) in order to identify potentially affected steroid hormones and their metabolizing enzymes. Further, we aimed to assess whether the mRNA expression of these steroid-metabolizing enzymes is detectable in the serum and, if so, whether mRNA expression of these enzymes is different between the study groups. Finally, we aimed to visualize these steroid-metabolizing enzymes postnatal in human placenta using immunohistochemical staining and to test whether levels of serum mRNA expression at 24 and 28 WGA might reflect postnatal placental protein expression.

## 2. Results

### 2.1. Clinical Characterization of the Study Population

Women of both groups were approximately at the same age, had a similar body mass index (BMI) and had the same number of experienced pregnancies (Table 1). In our cohort, women with preeclampsia had a significant higher rate of cesarean delivery (77.8%) than in the control group (38.9%). This reflects the urgency and complexity of managing affected women to minimize preeclampsia-related risks.

The gestational age of women with preeclampsia was on average 3 weeks shorter (*p* = 0.022) than in the control group, which resulted in a premature birth (i.e., birth at <37 WGA), which is typically observed in cases of preeclampsia and may have profound implications for neonatal outcomes. At the time of birth, the average weight of children of women with preeclampsia was 1108 g less than that of children of women in the control group (*p* = 0.004). Accordingly, the rate of children below the 10th growth percentile was significantly higher in women with preeclampsia (*p* = 0.001) than in controls, reflecting the impact of preeclampsia on fetal outcomes. 

Other parameters, such as obesity (BMI > 30) or prophylaxis with low-dose aspirin, did not differ significantly between both groups. Rates of hypertension, past preeclampsia and (systolic/diastolic) blood pressure (24–30 WGA) tended to be higher in the preeclampsia group, but these differences were not statistically significant. Almost two thirds of the controls did not perform a screening test for preeclampsia. The proportion of women without a screening test for preeclampsia was apparently (but not significantly) lower in the preeclampsia group.

### 2.2. Levels of Steroid Hormones in Serum 

**Figure 1:**
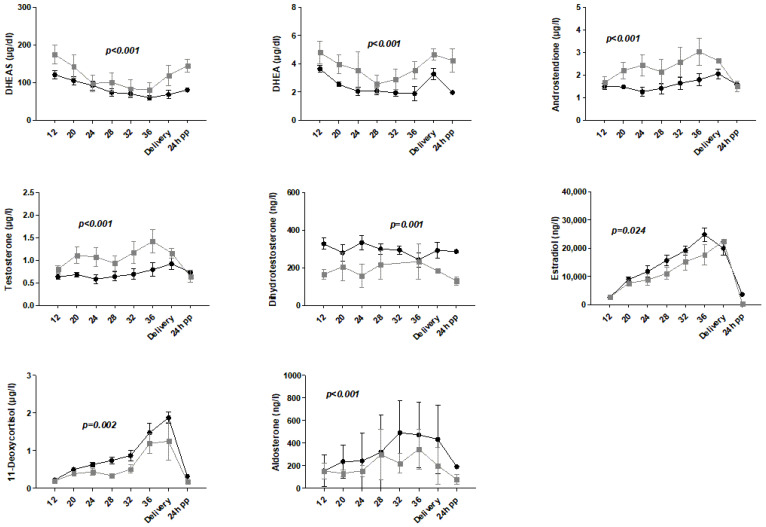
Concentration versus time course of DHEAS, DHEA, androstenedione, testosterone, dihydrotestosterone, estradiol, 11-deoxycortisol and aldosterone in women with preeclampsia (gray line with squares; mean ± SEM) and controls (black line with circles; mean ± SEM). *X*-axis: time (12, 20, 24, 28, 32 and 36 WGA, day of delivery and 24 h postpartum). Steroid comparisons between preeclamptic and healthy groups were calculated using Student’s *t*-test or Kruskal–Wallis test. The level of significance was 0.05. DHEAS, dehydroepiandrosterone sulfate; DHEA, dehydroepiandrosterone; pp, postpartum; SEM, standard error of the mean.

Mean androgen levels of dehydroepiandrosterone (DHEA), DHEAS, androstenedione and testosterone were significantly elevated, whereas mean levels of dihydrotestosterone (DHT), 17β-estradiol (estradiol), 11-deoxycortisol and aldosterone were significantly reduced in women with preeclampsia compared to controls (Figure 1).

**Figure 2:**
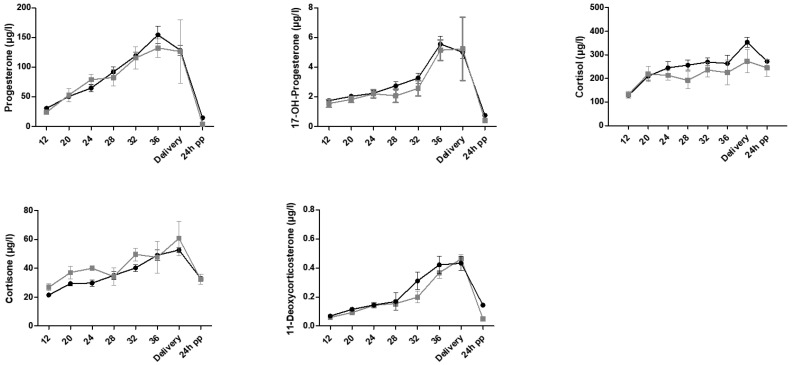
Concentration versus time course of progesterone, 17OH-progesterone, cortisol, cortisone and 11-deoxycorticosterone concentrations in women with preeclampsia (gray line with squares; mean ± SEM) and controls (black line with circles; mean ± SEM). *X*-axis: time (12, 20, 24, 28, 32 and 36 WGA, day of delivery and 24 h post-partum). Steroid comparisons between preeclamptic and healthy groups were calculated using Student’s *t*-test or Kruskal–Wallis test. The level of significance was 0.05. pp, post-partum; SEM, standard error of the mean.

The concentration versus time courses of the mean serum concentrations of progesterone, 17-OH progesterone (17-OHP), cortisol, cortisone and 11-deoxycorticosterone were similar between the women with preeclampsia and controls (Figure 2).

### 2.3. Precursor-to-Product Ratios of Steroid-Metabolizing Enzymes in Serum 

Based on longitudinal significant changes in precursor-to-product ratios in serum of women with preeclampsia, we identified potentially affected enzymatic candidate genes. 

**Figure 3:**
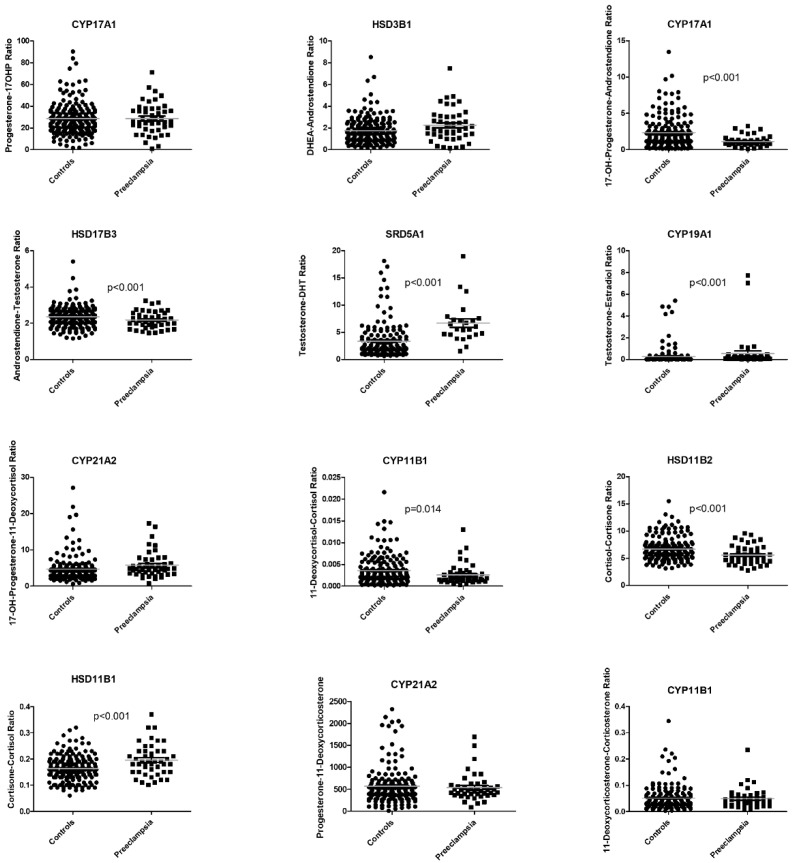
Boxplots of the precursor-to-product ratios of steroid-metabolizing enzymes in the preeclamptic and in control groups. Each point represents an observation during pregnancy at 12, 20, 24, 28, 32 and 36 WGA. Not all women were followed up at all time points. The respective mean values are presented as a gray line. *CYP17A1*, steroid 17α-hydroxylase; *HSD3B1*, 3β-hydroxysteroid-dehydrogenase; *HSD17B3*, 17β-hydroxysteroid-dehydrogenase; *SRD5A1*, 5α-reductase; *CYP19A1*, aromatase; *CYP21A2*, steroid 21-hydroxylase; *CYP11B1*, steroid 11β-hydroxylase; *HSD11B2*, 11β-hydroxysteroid dehydrogenase 2; *HSD11B1*, 11β-hydroxysteroid dehydrogenase 1; *CYP21A2*, steroid 21-hydroxylase; *CYP11B1*, steroid 11β-hydroxylase.

The precursor-to-product ratios of the steroid-metabolizing enzymes 5α-reductase (*SRD5A1*), aromatase (*CYP19A1*) and 11β-hydroxysteroid dehydrogenase 1 (*HSD11B1*) were significantly increased, whereas the precursor-to-product ratios of steroid 17-α-hydroxylase (*CYP17A1*), 17β-hydroxysteroid dehydrogenase (*HSD17B3*), steroid 11β-hydroxylase (*CYP11B1*) and 11β-hydroxysteroid dehydrogenase 2 (*HSD11B2*) were significantly decreased (Figure 3). All metabolized steroids, respective precursors and products and their metabolizing enzymes are presented in Table 2. The genes of these enzymes were selected as candidate genes for serum mRNA expression profiling and immunohistochemical staining of the placenta in all sera of the study cohort during WGA 26 and 28. 

### 2.4. Serum mRNA Expression of Selected Steroid-Metabolizing Enzymes During WGA 26 and 28 

mRNA expression of all examined steroid-metabolizing enzymes was detected in serum samples from pregnant women during WGA 26 and 28. During this period, the serum mRNA expression of steroid-metabolizing enzymes was not altered in women with preeclampsia compared to controls.

**Figure 4:**
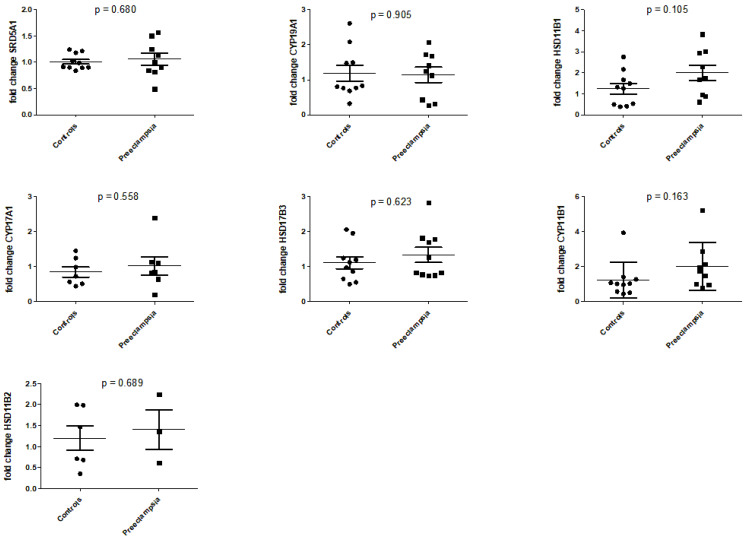
mRNA expression of steroid-metabolizing enzymes in serum of women with preeclampsia and of controls at WGA 24 and 28. Each dot represents an available sample of the 9 enrolled preeclamptic women and 36 healthy controls at WGA 24 and 28. *SRD5A1*, 5α-reductase; *CYP19A1*, aromatase; *HSD11B1*, steroid 11β-hydroxylase; *CYP17A1*, steroid 17α-hydroxylase; *HSD17B3*, 17β-hydroxysteroid-dehydrogenase; *CYP11B1*, steroid 11β-hydroxylase; *HSD11B2*, 11β-hydroxysteroid dehydrogenase 2.

mRNA expressions of genes of enzymes with significantly decreased precursor-to-product ratios (i.e., *CYP17A1*, *HSD17B3*, *CYP11B1* and *HSD11B2*) was similar in women with preeclampsia and controls, but the RNA levels of these genes were higher in women with preeclampsia (Figure 4). In the preeclampsia group, the mRNA expression of only one enzyme, namely *HSD11B1*, was directly proportional to its precursor-to-product ratio in serum.

### 2.5. Placental Protein Expression of Selected Steroid-Metabolizing Enzymes

The enzymes *SRD5A1*, *CYP11B1*, *HSD11B1* and *HSD11B2* were detected in various placental structures such as the syncytiotrophoblast, the endothelium, the extravillous trophoblasts or the decidua. The enzyme *CYP17A1* was not detected in any structure of the placenta. No differences were observed visually in the placental expression of the analyzed enzymes between women with preeclampsia and controls. Statistical analysis of the semi-quantified data also showed no differences in the placenta subgroup analysis between early- and late-onset preeclampsia (Table 3).

### 2.6. mRNA Expression of Selected Steroid-Metabolizing Enzymes at WGA 24 and 28

mRNA expression of steroid-metabolizing enzymes in serum did not reflect postnatal protein expression in the placenta. Table 4 provides an overview of serum steroid precursor-to-product ratios, mRNA expression profiles in serum and protein expression in the placenta. Subgroup analysis between women with early-onset preeclampsia (n = 3) and women with late-onset preeclampsia (n = 3) subsequently also showed no differences in placental steroid-metabolizing enzyme expression.

### 2.7. Placental Protein Expression of HSD11B1 and HSD11B2

**Figure 5:**
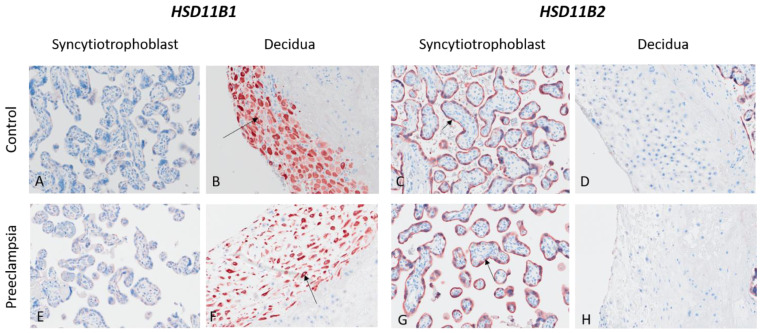
Placental protein expression of *HSD11B1* and *HSD11B2* in women with preeclampsia and controls. Epitopes of *HSD11B1* and *HSD11B2* were stained in red. *HSD11B1* was expressed in the cytoplasm of maternal decidua cells (black arrow in (**B**,**F**)), while *HSD11B2* was expressed in the fetal syncytiotrophoblast. The placental tissue shows uninterrupted staining on the outer surface of the villous tree (black arrow in (**C**,**G**)). There is no labelling for *HSD11B1* in the syncytiotrophoblast (**A**,**E**) and no visualization of *HSD11B2* in decidua cells (**D**,**H**).

*HSD11B2* was moderately to highly expressed (2.5) in the syncytiotrophoblast (fetal origin) of all women, while *HSD11B1* was expressed (2.35 ± 0.26) in the decidua (maternal origin) of all placentas (Figure 5).

The placental tissue shows uninterrupted staining on the outer surface of the villous tree (black arrow in C and G). There is no labelling for *HSD11B1* in the syncytiotrophoblast (A and E) and no visualization of *HSD11B2* in decidua cells (D and H).

## 3. Discussion

In our study serum levels of progesterone, estrogens, cortisol and aldosterone increased significantly, as expected during normal pregnancy, while DHEAS decreased slightly. We demonstrated that women with preeclampsia have altered steroid hormone levels throughout pregnancy compared to controls (healthy pregnant women), whereby sex hormones and glucocorticoids were particularly affected. We identified *CYP17A1*, *HSD17B3*, *SRD5A1*, *CYP19A1*, *CYP11B1*, *HSD11B1* and *HSD11B2* as potentially affected enzymes in preeclampsia. These enzymes did not show preeclampsia-related differences in their serum mRNA levels at the end of the second trimester of pregnancy. Also, placental protein expression did not show any differences between women with preeclampsia and controls. Postnatal protein expression in the placenta may therefore not be reflected by the mRNA expression of steroid-metabolizing enzymes in serum from WGA 24 to WGA 28. 

Our data support several experimental and clinical findings: 

First, women with preeclampsia have even higher levels of circulating lipids [24,25,26] than the already elevated plasma lipid levels that are typical features of healthy pregnancy. Increased availability of cholesterol in steroid hormone-producing cells, e.g., due to obesity, may affect the pregnancy-associated regulation of steroid hormones, which occurs at the level of gene transcription for the steroidogenic enzymes. This can, in consequence, lead to increased synthesis capacity of the cells [26] and to alterations in serum steroid profiles during the development of preeclampsia. In our dataset, progesterone levels showed a similar course in preeclamptic women and controls (*p* = 0.904). The progesterone-17-OHP ratio, representing *CYP17A1* activity in the zona reticularis of the adrenal cortex, exhibited no changes in preeclamptic women. By contrast, the 17-OHP-to-androstenedione ratio (both OHP and androstenedione are metabolized by the enzyme *CYP17A1* in the testes, ovary or placenta) seems to be affected by preeclampsia, defined by a reduced ratio (*p* < 0.001). These findings suggest that steroid metabolism in women with preeclampsia is disturbed at a later phase in the pathway, such as in the zona glomerulosa or the zona fasciculata of the adrenal cortex, the gonadal organs or the placenta.

Second, previous studies have indicated that controlling angiogenesis by gonadal hormones is fundamental for uterine changes throughout pregnancy [27]. During gestation, estrogen is produced primarily in the placenta through the conversion of androgen precursors originating from maternal and fetal adrenal glands. These processes lead to increased estrogen levels compared to those in non-pregnant women [28]. Since there is a considerable role of estrogen in maintaining uteroplacental vascular function, a decrease in estrogen levels may play a key role in the development of preeclampsia. Our data support several clinical studies that have been showing a significant decrease in estradiol levels during preeclampsia at one to three visits during pregnancy [29,30,31,32]. 

The continuous monitoring of estrogen levels every four weeks throughout the pregnancy in our study (eight visits in total) provided a comprehensive picture of the development of preeclampsia over time. This revealed that the decline in estradiol levels began at around the 12th week of pregnancy and continued until at least 24 postpartum. Accordingly, the exogenous administration of estrogen has been shown to produce beneficial effects in animal models as well as in preeclampsia patients [29,33]. 

Women with polycystic ovary syndrome, who have typically higher androgen levels, are at higher risk for preeclampsia [34]. Notably, the testosterone-to-estradiol ratio, which indicates altered availability and/or function of *CYP19A1*-encoded aromatase was significantly different between women with preeclampsia and controls in our study. This is consistent with the finding that androstenedione and testosterone levels were increased, and estradiol levels were decreased in women with preeclampsia compared to controls. The conversion of androstenedione-to-testosterone, catalyzed by *HSD17B3*, was enhanced (expressed in a decreased ratio) in preeclamptic women.

In the next step of the gonadal steroid metabolism catalyzed by *SRD5A1*, the ratio of testosterone to DHT was significantly increased in preeclamptic women compared to controls, which may be a consequence of the reduced conversion of testosterone to DHT in the previous step in preeclampsia. In the conversion of DHEA to androstenedione by *HSD3B1*, both metabolites were elevated in preeclampsia. Their ratio tended to be increased in preeclampsia, indicating that increase of DHEA may be higher than that of androstenedione in women with preeclampsia. These observations are consistent with the placental expression of *SRD5A1* in our data. Women with preeclampsia showed lower semi-quantitative scores for the stained placental structures than controls, but due to the small sample size, data did not reach statistical significance. Sex steroid-metabolizing enzymes appear to be affected in women with preeclampsia, which might contribute to the hormonal imbalances of steroids associated with preeclampsia.

Third, cortisol availability in the body is controlled by the enzyme *HSD11B2*, encoded by the respective gene, which inactivates cortisol to the metabolite cortisone and thereby protecting the fetus from excessive maternal stress exposure [35,36]. The *HSD11B2* enzyme activity limits either intracellular cortisol concentrations or—within the uteroplacental compartment—the transfer of cortisol into the fetal circulation. The *HSD11B1* gene acts reversely to *HSD11B2* and reactivates cortisol by converting cortisone to cortisol [35].

Consistent with results from Jayasuriya et al. [36], our data showed that the maternal cortisol-to-cortisone ratio in women with preeclampsia was already reduced by 20 WGA. This difference remained present until the time of giving birth and returned to the ratio of controls only 24 h postpartum. Our mRNA expression data showed a tendency towards higher *HSD11B2* mRNA levels in the serum of women with preeclampsia, whereas placental *HSD11B2* mRNA expression (and activity) has been found to be reduced in women with preeclampsia [35,37,38].

In our placental studies, the *HSD11B2* protein was expressed mainly in the fetal syncytiotrophoblasts, whereas *HSD11B1* was detected in maternal decidua cells, suggesting that placental inactivation of cortisol to cortisone is mainly induced by the fetus, whereas activation of cortisone to cortisol is induced by the mother. However, the significance of these results is limited by the small number of additional placentas for the preeclampsia group (n = 3) for these analyses.

Interestingly, levels of serum 11-deoxycortisol, the precursor of cortisol, were significantly decreased (*p* = 0.002) in women with preeclampsia already at an earlier step in the metabolism of glucocorticoids. Further, the 17OHP-to-11-deoxycortisol ratio, metabolized by *CYP21A2*, tended to be increased (*p* = 0.059), indicating that enzymes in earlier steps of the glucocorticoid metabolism (*CYP11B1* and *CYP21A2*) may already be affected. Another explanation could be that this decrease may be affected by renal dysfunction as seen in preeclampsia [39].

mRNA as a systemically circulating nucleic acid has been reviewed by Carbone et al. in 2020 for potential applications in pregnancy disorders such as preeclampsia [23]. It has been proposed that multiple subtypes/pathophysiological processes of preeclampsia are leading to common clinical presentations [40]. Therefore, several efforts have focused on molecular subtyping of preeclampsia by performing genome-wide mRNA expression microarray studies [41,42,43,44]. Some of the identified differently expressed genes include genes related to steroid metabolism, such as the cholesterol monooxygenase (*CYP11A1*) [43] or hydroxysteroid 17β–dehydrogenase 1 (*HSD17B1*) [44], highlighting the importance of steroid metabolism in preeclampsia.

However, published expression data of the present candidates are sparse, both in the placenta [30] and all the more so in blood, where we did not identify any published studies. Systemic mRNA expression exploration of the investigated steroid-metabolizing enzymes by the Human Protein Atlas (HPA) program (www.proteinatlas.org, accessed on 29 January 2024) revealed that *SRD5A1* is mainly expressed in the liver, skin and breast. *HSD3B1* is highly expressed in the placenta and the small intestine, whereas *CYP11B1* is primarily expressed in the adrenal gland. Shin et al. have detected reduced placental *HSD3B1* mRNA expression in women with preeclampsia [30], which is consistent with the increased serum DHEA-to-androstenedione ratio in our study cohort. Further, we detected a non-significantly lower HSDB1 protein expression in syncytiotrophoblast and extravillous trophoblasts, suggesting that this enzyme is affected by lower mRNA expression and translation but not by reduced protein activity. Notably, all enzymes are also expressed in several immune cell types and the peripheral blood mononuclear cells (PBMCs) in the Monaco dataset [45] and the Schmiedel dataset [46], pointing out the heterogeneity of these pathways. 

Our study has some limitations. The expression data are limited by the small group sample sizes. Serum precursor-to-product ratios may therefore not be conclusively confirmed by mRNA concentrations of steroid-metabolizing enzymes as well as by placental protein expression. Further, not all women attended their eight follow-up appointments for various reasons. Due to the small sample size in our groups, we are aware of several other limitations, namely limited generalizability, risk of random variation and limited exploration of heterogeneity. In rare conditions like preeclampsia, small sample sizes may also fail to capture the diversity of clinical presentations, limiting applicability to subgroups, or be confounded by other related factors such as gestational differences. Sample sizes at individual time points were too small to provide statistically robust data. By consolidating the data across the entire pregnancy, the analysis could be conducted with a sufficient sample size, enabling more reliable and meaningful conclusions. However, levels of steroids and their precursors may vary considerably depending on factors such as the time of the onset of preeclampsia, the severity of the disease and the individual characteristics of each patient, which were not taken into account in this study. We are aware that conclusions about biomarkers could be affected by limitations in placental mRNA expression. If the expression of the investigated steroid-metabolizing enzymes is unintentionally restricted (e.g., by sample handling or biological variability), this may result in the underestimation of biomarkers, misinterpretation of pathophysiological roles and/or reduced reproducibility. In subsequent research, sample sizes need to be increased to improve statistical power. Outcomes should be analyzed separately for important subgroups of preeclampsia such as late-onset and early-onset preeclampsia. A balanced study design should be used to reduce confounding.

Strengths of our study are the in-depth clinical and biochemical characterization of all pregnant women during pregnancy, which included a high number of study visits and the use of a state-of-the-art and standardized bioanalytical method to measure all steroid hormones presented. 

## 4. Materials and Methods

### 4.1. Patients and Methods

Out of a prospective longitudinal cohort study in 394 women with high-risk pregnancies, a subgroup of 9 women with preeclampsia and 36 pregnant women without known complications, referred to as controls, were selected. Details of this study have been published previously [47]. Briefly, women were recruited at the time of admission for prenatal care, starting between 10 and 12 WGA in a tertiary gynecological university center.

Preeclampsia and hypertension were defined according to [1,2]. Fetal growth restriction was defined as fetal growth < 5th percentile of gestational age. Early-onset preeclampsia was defined as an onset of preeclampsia < 34 WGA, and late-onset preeclampsia as an onset of preeclampsia > 34 WGA.

The first trimester screening for preeclampsia, if performed, consisted of a combination of maternal demographic characteristics (including medical and obstetric history), uterine artery pulsatility index (PI), mean arterial pressure (MAP), maternal serum pregnancy-associated plasma protein-A (PAPP-A), and placental growth factor (PlGF) at 11–13 WGA [48]. 

After enrolment, up to eight study visits were made at 12, 20, 24, 28, 32 and 36 WGA, on the day of delivery and 24 h postpartum for routine blood sampling. Blood samples without anticoagulant were centrifuged at 800× *g* for 10 min; sera were portioned into 200 μL aliquots and stored at −80 °C at the Biobank Graz, Austria [49]. PAXgene tubes (Qiagen, Hilden, Germany) were collected from women enrolled in the study between 10 June 2015 and 29 September 2017. PAXgene tubes were frozen at −20 °C within 4 h until RNA extraction. The study protocol was approved by the Medical University Ethics Committee (IRB00002556). Written informed consent was obtained from all participants.

### 4.2. Mass Spectrometry Measurement 

**Figure 6:**
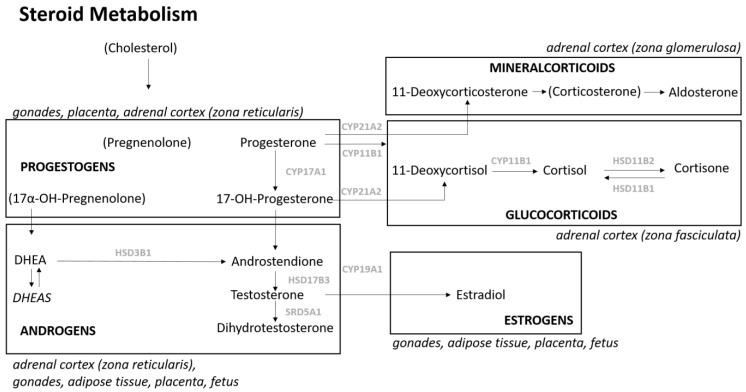
Steroid metabolism, adapted from [51]. Sources of the respective steroid class are shown in italics. Steroids in brackets were not measured in this study. Metabolizing-enzymes are shown in gray and are en-coded by the gene of the same name. *CYP17A1*, steroid 17α hydroxylase; *HSD17B3*, 17β-hydroxysteroid-dehydrogenase; *SRD5A1*, 5α-reductase; *CYP19A1*, aromatase; *HSD3B1*, 3β hydroxysteroid dehydrogenase; *CYP21A2*, steroid 21-hydroxylase; *CYP11B1*, steroid 11β-hydroxylase; *HSD11B2*, 11β-hydroxysteroid dehydrogenase 2, *HSD11B1*, 11β-hydroxysteroid dehydrogenase 1.

Liquid chromatography tandem mass spectrometry (LC-MS/MS) measurements were performed with a liquid chromatography tandem mass spectrometry system (QTRAP 6500+, SCIEX^®^, Framingham, MA, USA), including an Agilent 1290 HPLC (G4226A autosampler, infinityBinPump, G1316C column-oven, G1330B thermostat, Santa Clara, CA, USA). An IVDR conform kit MassChrom-Steroids in Serum/Plasma^®^ (Chromsystems^®^, Gräfelfing, Germany) was used for the quantification of 14 steroid hormones (Progesterone, 17OHP, DHEAS, DHEA, androstenedione, testosterone, dihydrotestosterone, estradiol, 21-deoxycortisol, 11-deoxycortisol, cortisol, cortisone, 11-deoxycorticosterone and aldosterone) [50] in MRM mode via corresponding isotope-labeled standards according to the manufacturer’s instruction. A volume of 500 μL serum was processed by offline solid phase extraction, and 15 μL were used for analysis. Quantitative analysis was performed with the Analyst^®^ Software (1.6.3), SCIEX®, Framingham, MA, USA via 6-point calibration, and 1/x weighting was used. Correctness of measurements was ensured by commercial quality controls and periodic participation in ring trails. Selected steroid hormones and their role in steroid metabolism are shown in Figure 6.

### 4.3. RNA Extraction and qPCR

For qPCR analysis, samples from visit 3 (24 WGA) and visit 4 (28 WGA) were analyzed and shown in a combined boxplot. These time points were selected because angiogenic factors (sFlt-1 and PlGF) were shown to be altered in women later developing early-onset preeclampsia (defined as delivery < 32 WGA) already about five weeks prior to onset of clinical symptoms [52].

RNA was isolated using a PAXgene Blood RNA Kit (Qiagen, Hilden, North Rhine-Westphalia, Germany) according to the manufacturer’s instructions and was eluted from the columns by adding 25 μL RNase-free water, followed by centrifugation. RNA purity (260/280), as well as RNA yield, were measured using a Nanodrop (Thermo Fisher Scientific, Waltham, MA, USA). The isolated RNA was stored at −80 °C for the short term. Complementary DNA (cDNA) was generated using a High-Capacity cDNA Reverse Transcription Kit (Thermo Fisher Scientific, Waltham, MA, USA), and subsequent quantitative real-time PCR (qPCR) was performed in duplicates using TaqMan MRNA expression MasterMix and TaqMan MRNA expression Assays on demand by Applied Biosystems by Thermo Fischer Scientific (Waltham, MA, USA) with a CFX384 Touch Real-Time PCR Detection System (Bio-Rad, Hercules, CA, USA). The relative expression levels of all investigated mRNAs were calculated as fold change [53]. Therefore, average Ct values have been normalized to the mean of beta-actin and protein phosphatase 1 regulatory subunit 15B (PPP1R15B) used as housekeeping genes [54]. Fold change was calculated as 2^−ΔΔCt^, where ΔΔCt was ΔCt of preeclampsia patients minus ΔCt of controls. Quantitative qPCR data were reported as mean ± standard deviation (SD).

### 4.4. Placenta Tissue Collection

To investigate placental enzyme expression, we selected samples from a separate collection of formalin-fixed paraffin-embedded (FFPE) placental tissue blocks from pre-eclamptic women (n = 6) and controls (n = 6).

### 4.5. Immunohistochemistry

The following target proteins were stained using polyclonal rabbit antibodies (Sigma-Aldrich, Darmstadt, Germany): 17-α-hydroxylase(*CYP17A1*), 17β-hydroxysteroid dehydrogenase (*HSD17B3*), steroid 5α-reductase (*SRD5A1*), 11β-hydroxysteroid dehydrogenase 1, (*CYP11B1*), 11β-hydroxysteroid dehydrogenase 2 (*HSD11B2*) and 11β-hydroxysteroid dehydrogenase 1 (*HSD11B1*).

Sections of FFPE placenta tissue blocks (thickness: 5 µm) were deparaffinized and rehydrated according to standard procedures. Antigen retrieval was conducted in a microwave oven in Tris-EDTA buffer, pH 9, for 40 min. After washing with TBS including 0.05% Tween (Merck, Darmstadt, Germany) (TBS/T), sections were incubated with Hydrogen Peroxide Block (Epredia, Breda, Hovedstaden, The Netherlands) to quench endogenous peroxidase. A further washing step with TBS/T was followed by blocking nonspecific background with UltraVision Protein Block (Epredia, Hovedstaden Breda, The Netherlands). The antibodies (rabbit, polyclonal antibody, Sigma-Aldrich, Darmstadt, Germany) #HPA048533 (*CYP17A1*), #HPA056833 (*HSD17B3*), #HPA051402 (*SRD5A1*), #HPA056348 (*CYP11B1*), #HPA056385 (*HSD11B2*) and #HPA047729 (*HSD11B1*) were diluted for 1:100, 1:200, 1:500 or 1:1000 in Antibody Diluent (Agilent Dako, Santa Clara, CA, USA), and sections incubated for 45 min at room temperature (RT). Slides were then washed with TBS/T and incubated with an HRP-labelled polymer (Epredia, Breda, Hovedstaden, The Netherlands) for 20 min at RT. Following another washing step, the polymer complex was visualized with AEC (AEC Substrate Kit, Abcam, Cambridge, UK), and sections were counterstained with hemalaun and mounted with Kaiser’s glycerin gelatine (Merck, Darmstadt, Germany). Slides were scanned using an Olympus VS200 slide scanner (Olympus, Hamburg, Germany).

Protein expression of the steroid-metabolizing enzymes was assessed by evaluating the staining in the following placental structures: syncytiotrophoblast, villous cytotrophoblast, endothelium, macrophages, stroma, extravillous trophoblast and decidua. These placental structures were visually scored semi-quantitatively by a histological expert in the field, including staining intensity from—(no protein expression) to +++ (high expression) and percentage of stained cells from—(no cell) to +++ (all cells), then converted into a numerical score.

### 4.6. Statistical Analysis

Statistical analysis was performed using SPSS statistics version 25.0 (IBM SPSS Statistics GmbH, Baden-Württemberg, Germany). Patient characteristics and biomarker results were reported as mean ± SD unless otherwise stated.

To assess differences in the concentration-time curves, we tested for differences between the overall means during pregnancy using Student’s *t*-test (parametric data) and Kruskal–Wallis test (non-parametric data). The distribution of data was analyzed using descriptive statistics and the Kolmogorov–Smirnov test, as well as by evaluating quantile–quantile plots. Normally distributed quantitative data were compared using unpaired Student’s *t*-test. To assess the assumption of homogeneity of variances, Levene’s test was performed. When Levene’s test indicated significant variance inequality, we performed Welch’s *t*-test that adjusts degrees of freedom to account for unequal variances, ensuring robust comparisons between groups. Unequally distributed data were compared by applying Kruskal–Wallis tests for non-parametric samples. 

Semi-quantitative values of each placental structure were compared between both study groups using Student’s *t*-test.

Observed frequencies for categorical data were compared using the Chi-square (Χ^2^) test. Changes of RNA levels in the preeclampsia group are displayed as relative change compared to RNA levels of healthy controls as reference. A *p*-value of ≤0.05 was considered statistically significant.

## 5. Conclusions

Serum steroid levels were significantly altered in preeclamptic women compared to controls during pregnancy. Following significantly altered precursor-to-product ratios, the respective enzymes *CYP17A1*, *HSD17B3*, *SRD5A1*, *CYP19A1*, *CYP11B1*, *HSD11B1* and *HSD11B2* are potentially affected steroid-metabolizing enzymes that are contributing to the imbalances of steroid hormones. All of the abovementioned disturbances might potentially be associated with the pathogenesis of preeclampsia. The disturbance of steroid metabolism with regard to the gonadal hormones appeared to take place only at the level of metabolizing stages after the typical changes in the reticulate zone of the adrenal gland, the reproductive organs and the placenta. With regard to glucocorticoids and mineral corticoids, the disruption of steroid metabolism seemed to occur in the glomerular and fascicular zone of the adrenal gland, but not in the zona reticularis. Genes of these steroid-metabolizing enzymes were expressed in the serum of pregnant women. However, they did not show preeclampsia-related differences in their serum and placental expression in additional placenta samples of pregnant women with and without preeclampsia. The trend of the results from mRNA expression profiles of the investigated steroid-metabolizing enzymes in serum did not reflect the protein expression in the placenta itself. 

Steroid-metabolizing enzymes should be further investigated in larger cohorts to define a possible involvement in the pathogenesis of preeclampsia. Insights into circulating RNA profiles such as mRNAs could provide a sound foundation for the pathophysiological understanding of the heterogeneity of preeclampsia and could therefore be of importance for future research in personalized medicine and diagnostic treatment strategies for women with preeclampsia.

## Figures and Tables

**Table 1:** Demographic and clinical characteristics of the study population.

	Women with Preeclampsia	Controls	*p*-Value
N	9	36	
Age (y)	32.3 ± 4.4	31.9 ± 4.9	0.971
BMI (kg/m^2^)	31.1 ± 8.6	28.0 ± 7.8	0.969
Gravidity [n]	2.2 ± 1.2	2.8 ± 1.8	0.316
Mode of delivery [vaginal ^#^/C-section]	2/7	19/14	0.06
Systolic blood pressure (mmHg) *	134.1 ± 16.8	127.1 ± 13.5	0.234
Diastolic blood pressure (mmHg) *	87.9 ± 13.8	78.5 ± 11.1	0.306
Week of pregnancy at delivery (weeks)	35.7 ± 2.7	38.7 ± 2.9	0.022
Early-onset PE (<34 WGA), [n (%)]	3 (33.3)	n.a.	
Late-onset PE (>34 WGA), [n (%)]	6 (66.7)	n.a.	
Birthweight (g)	2267.0 ± 871.7	3375.7 ± 468.0	0.004
Fetal sex (male/female)	4/5	21/12	0.158
Growth percentile ≤ 10 [n (%)]	5 (55.6)	3 (8.8)	0.001
Obesity (BMI > 30) [n (%)]	4 (44.4)	10 (28.6)	0.362
Hypertension [n (%)]	5 (55.6)	9 (25.7)	0.086
History of former preeclampsia [n (%)]	4 (44.4)	6 (17.1)	0.081
Prophylaxis with low-dose aspirin treatment [n (%)]	7 (77.8)	17 (48.6)	0.117
Screening for preeclampsia:			0.081
Not performed [n (%)]	4 (44.4)	23 (67.6)	
Inconspicuous [n (%)]	1 (11.1)	7 (20.6)	
Suspicious [n (%)]	4 (44.4)	4 (11.8)	

Values are shown as mean (SD); y, year; n, number; kg, kilograms; m^2^, square meter; mmHg, millimeters of mercury; PE, preeclampsia; %, percent; BMI, body mass index; n.a., not analyzed. ^#^ Spontaneous vaginal delivery and assisted vaginal delivery; * values were shown from 24–30 WGA, representative of pregnancy-related changes but not yet late pregnancy.

**Table 2:** Steroid-metabolizing enzymes, precursors, products and precursor-to-product ratios of steroid-metabolizing enzymes in women with preeclampsia and in controls.

Steroid-Metabolizing Enzymes	Assay ID	Precursor	to	Product	Ratio in Women with PE	Ratio in Controls	Direction of Ratio in Women with Preeclampsia	*p*-Value	Observed GE in Women with PE
Progestogens									
*CYP17A1*		Progesterone	→	17-OH-Progesterone	29.04 ± 15.33	28.73 ± 14.51		0.904	
Sex steroids									
*HSD3B1*	HS04194787_g1	DHEA	→	Androstenedione	2.15 ± 1.51	1.76 ± 1.19		0.081	
*CYP17A1*	HS01124136_m1	17-OH-Progesterone	→	Androstenedione	1.11 ± 0.70	2.33 ± 2.07	↓	<0.001	↑
*HSD17B3*	Hs00609319_m1	Androstenedione	→	Testosterone	2.18 ± 0.44	2.35 ± 0.56	↓	<0.001	
*SRD5A1*	HS00602694	Testosterone	→	Dihydrotestosterone	6.69 ± 3.82	3.36 ± 3.24	↑	<0.001	⊥
*CYP19A1*	HS00903411_m1	Testosterone	→	Estradiol	0.61 ± 1.64	0.55 ± 2.33	↑	<0.001	⊥
Glucocorticoids									
*CYP21A2*		17-OH-Progesterone	→	11-Deoxycortisol	5.86 ± 3.65	4.73 ± 3.39		0.059	
*CYP11B1*	HS00357016_g1	11-Deoxycortisol	→	Cortisol	0.0027 ± 0.0026	0.0036 ± 0.0031	↓	0.014	↑
*HSD11B2*	HS01547870_m1	Cortisol	→	Cortisone	0.18 ± 0.06	0.19 ± 0.07	↓	<0.001	↑
*HSD11B1*	HS00388669_m1	Cortisone	→	Cortisol	0.20 ± 0.06	0.16 ± 0.05	↑	<0.001	↑
Mineralocorticoids									
*CYP21A2*		Progesterone	→	11-Deoxycorticosterone	562 ± 326	568 ± 419		0.659	
*CYP11B1*		11-Deoxycorticosterone	→	Corticosterone	0.05 ± 0.04	0.05 ± 0.05		0.546	

Significant changes in precursor-to-product ratios are in bold. →, is converted to; ↑, increased ratio; ↓, decreased ratio; ⊥, consistent ratio between the two groups. GE, mRNA expression; *CYP11B1*, steroid 11β-hydroxylase; *CYP17A1*, steroid 17α hydroxylase; *SRD5A1*, 5α-reductase; *CYP21A2*, steroid 21-hydroxylase; *HSD11B1*, 11β-hydroxysteroid dehydrogenase 1, *HSD11B2*, 11β-hydroxysteroid dehydrogenase 2; *CYP19A1*, aromatase; *HSD3B1*, 3β hydroxysteroid dehydrogenase; *HSD17B3*, 17β-hydroxysteroid-dehydrogenase. Precursor-to-product ratio comparisons between preeclamptic and healthy women were calculated using Student’s *t*-test or Kruskal–Wallis test. The level of significance was 0.05.

**Table 3:** A semi-quantitative numerical score of placental steroid-metabolizing enzymes.

Enzymes	Preeclampsia (n = 6)	Control (n = 6)	*p*-Value
	Syncytiotrophoblast		
*SRD5A1*	0.33 ± 0.20	0.46 ± 0.19	0.296
*CYP11B1*	0.33 ± 0.26	0.46 ± 0.33	0.484
*HSD11B2*	2.5	2.5	*
*HSD11B1*	0.42 ± 0.30	0.58 ± 0.47	0.479
*CYP17A1*	not detected	not detected	not detected
	Endothelium		
*SRD5A1*	0.29 ± 0.25	0.33 ± 0.20	0.756
*CYP11B1*	0.33 ± 0.26	0.29± 0.10	0.721
*HSD11B2*	0	0	*
*HSD11B1*	0.08 ± 0.13	0.29 ± 0.25	0.096
*CYP17A1*	not detected	not detected	not detected
	Extravillous Trophoblast		
*SRD5A1*	0.29 ± 0.10	0.17 ± 0.20	0.209
*CYP11B1*	0.10 ± 0.14	0.21 ± 0.19	0.313
*HSD11B2*	0	0	*
*HSD11B1*	0.10 ± 0.14	0.46 ± 0.43	0.101
*CYP17A1*	not detected	not detected	not detected
	Decidua		
*SRD5A1*	0	0.20 ± 0.29	0.272
*CYP11B1*	0.19 ± 0.13	0.29 ± 0.29	0.526
*HSD11B2*	0	0	*
*HSD11B1*	2.37 ± 0.25	2.33 ± 0.26	0.807
*CYP17A1*	not detected	not detected	not detected

Data are shown as median ± standard deviation; *, no difference.

**Table 4:** Precursor-to-product ratios, mRNA expression profiles in serum and protein expression in the syncytiotrophoblast of the placenta.

Gene/Enzyme	Trend Direction of Ratio in Preeclampsia *	Trend Direction of Serum mRNA Expression **	Trend Direction of Placental Protein Expression in the in the Syncytiotrophoblast **
*HSD3B1*	↑	Ʇ	↓
*CYP17A1*	↓	↑	n.a.
*HSD17B3*	↓	↑	
*SRD5A1*	↑	Ʇ	↓
*CYP11B1*	↓	↑	↓
*HSD11B2*	↑	↑	Ʇ
*HSD11B1*	↓	↑	↓

* the comparison between the groups was statistically significant (*p* ≤ 0.05). ** the comparison between the groups was not statistically significant (*p* > 0.05). ↑, increasing direction; ↓, decreasing direction; Ʇ, no direction; n.a., not analyzed. *SRD5A1*, 5α-reductase; *HSD3B1*, 3β-hydroxysteroid dehydrogenase; *CYP17A1*, steroid 17α-hydroxylase; *CYP11B1*, steroid 11β-hydroxylase; *HSD11B1*, 11β-hydroxysteroid dehydrogenase 1; *HSD11B2*, 11β-hydroxysteroid dehydrogenase 2.

## Data Availability

Data generated or analyzed during this study are included in this published article.
